# Supplementary material for: The causal relationship between the human gut microbiota and pyogenic arthritis: a Mendelian randomization study
Source: Front Cell Infect Microbiol. 2024 Nov 26;14:1452480. doi: 10.3389/fcimb.2024.1452480 (PMC11629706; doi:10.3389/fcimb.2024.1452480)
Supplement: Supplementary file 1 [file DataSheet1.zip › mendelian test/ebi-a-GCST90027605.csv_forest.pdf]

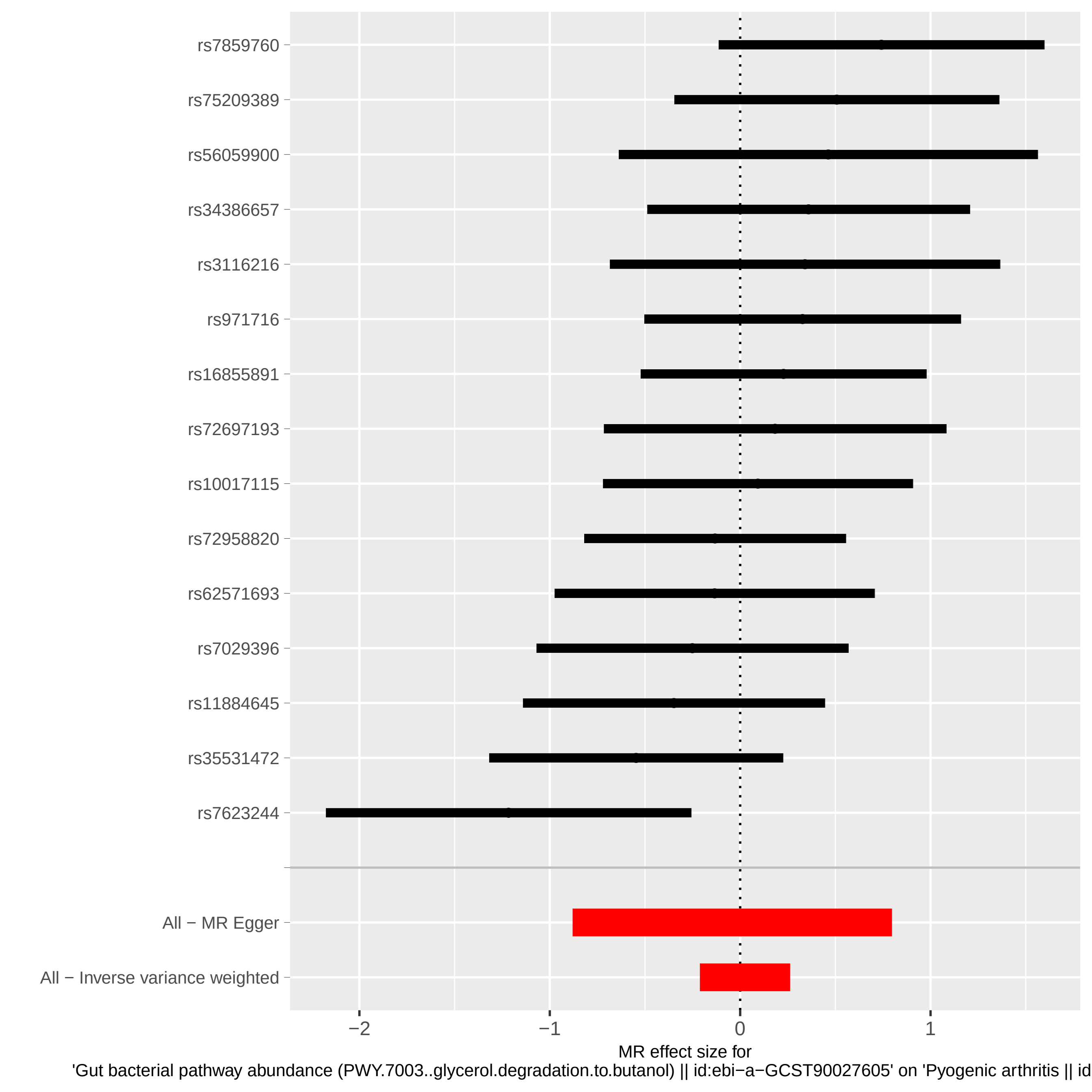
rs7859760
rs75209389
rs56059900
rs34386657
rs3116216
rs971716
rs16855891
rs72697193
rs10017115
rs72958820
rs62571693
rs7029396
rs11884645
rs35531472
rs7623244
All − MR Egger
All − Inverse variance weighted
−2
−1
0
1
MR effect size for
'Gut bacterial pathway abundance (PWY.7003..glycerol.degradation.to.butanol) || id:ebi−a−GCST90027605' on 'Pyogenic arthritis || id
